# Reviewing the role of healthy volunteer studies in drug development

**DOI:** 10.1186/s12967-018-1710-5

**Published:** 2018-12-04

**Authors:** Joyson J. Karakunnel, Nam Bui, Latha Palaniappan, Keith T. Schmidt, Kenneth W. Mahaffey, Briggs Morrison, William D. Figg, Shivaani Kummar

**Affiliations:** 1 Arcus Biosciences, Inc., 3928 Point Eden Way, Hayward, CA 94545 USA; 2 Stanford Cancer Institute, 875 Blake Wilbur Drive, Stanford, CA 94305 USA; 3 Department of Medicine, Stanford University School of Medicine, 900 Blake Wilbur Drive, Room W200, 2nd Floor MC 5358, Stanford, CA 94304 USA; 4 Clinical Pharmacology Program, Center for Cancer Research, National Cancer Institute, NIH, Bethesda, MD 20892 USA; 5 Stanford Center for Clinical Research (SCCR), Department of Medicine, Stanford University School of Medicine, 300 Pasteur Drive, Grant S-102, Stanford, CA 94305 USA; 6 Syndax, 211 East 43rd Street, #900, New York, NY 10017 USA

**Keywords:** Healthy volunteer, Phase 1, First-in-human, Safety, Toxicity, Pharmacokinetic, Pharmacodynamic, Bioequivalence, Medical ethics

## Abstract

**Background:**

With the exception of genotoxic oncology drugs, first-in-human, Phase 1 clinical studies of investigational drugs have traditionally been conducted in healthy volunteers (HVs). The primary goal of these studies is to investigate the pharmacokinetics and pharmacodynamics of a novel drug candidate, determine appropriate dosing, and document safety and tolerability.

**Main body:**

When tailored to specific study objectives, HV studies are beneficial to manufacturers and patients alike and can be applied to both non-oncology and oncology drug development. Enrollment of HVs not only increases study accrual rates for dose-escalation studies but also alleviates the ethical concern of enrolling patients with disease in a short-term study at subtherapeutic doses when other studies (e.g. Phase 2 or Phase 3 studies) may be more appropriate for the patient. The use of HVs in non-oncology Phase 1 clinical trials is relatively safe but nonetheless poses ethical challenges because of the potential risks to which HVs are exposed. In general, most adverse events associated with non-oncology drugs are mild in severity, and serious adverse events are rare, but examples of severe toxicity have been reported. The use of HVs in the clinical development of oncology drugs is more limited but is nonetheless useful for evaluating clinical pharmacology and establishing an appropriate starting dose for studies in cancer patients. During the development of oncology drugs, clinical pharmacology studies in HVs have been used to assess pharmacokinetics, drug metabolism, food effects, potential drug–drug interactions, effects of hepatic and renal impairment, and other pharmacologic parameters vital for clinical decision-making in oncology. Studies in HVs are also being used to evaluate biosimilars versus established anticancer biologic agents.

**Conclusion:**

A thorough assessment of toxicity and pharmacology throughout the drug development process is critical to ensure the safety of HVs. With the appropriate safeguards, HVs will continue to play an important role in future drug development.

## Background

Clinical drug development is divided into 4 phases. Phase 1 studies are designed to establish the safety and tolerability profile of an investigational drug and the recommended Phase 2 dose [1–3]. Phase 2 studies are designed to establish the clinical effectiveness of a novel drug candidate in a small patient population at a therapeutic dose [1, 2]. Phase 3 studies are usually large, randomized, controlled trials designed to establish the benefit-risk profile of a novel drug candidate at the recommended dose and schedule and to support regulatory approval [1, 2]. Finally, Phase 4 studies are post-approval studies designed to further define the safety and effectiveness of an approved drug in a real-world setting [1, 2].

With the exception of genotoxic oncology drugs, first-in-human (FIH), Phase 1 clinical studies for a wide range of investigational drugs have traditionally been conducted in healthy volunteers (HVs), defined by the National Institutes of Health as “someone with no known significant health problems who participates in research to test a new drug, device, or intervention” [4]. The primary goal of HV studies is to investigate the pharmacokinetics (PK) and pharmacodynamics (PD) of a novel drug candidate, determine appropriate dosing, and document safety and tolerability [3]. Phase 1 trials typically involve 20 to 80 HVs divided into small cohorts of 3 to 6 subjects who receive escalating doses of the investigational drug. The goal is to determine the mechanisms by which the drug is absorbed, metabolized, and excreted; define the PK profile; and characterize the safety and tolerability profile in humans across a range of doses [1, 2]. In the United States, approximately 70% of experimental drugs pass the first phase [5]. The HV model is ideal for this type of early clinical research because it allows testing of the pharmacology and safety profile of a drug candidate without the influence of any pathological conditions.

HV studies typically include both male and female subjects. However, historically, women were underrepresented in clinical trials, particularly in early trials, or excluded due to pregnancy risks [6–8]. In the early 1990s, regulatory authorities requested the inclusion of women in drug development to thoroughly evaluate potential gender-related differences in the clinical pharmacology of new therapeutic agents [9]. Since then, it has been recognized that women and men differ in how they absorb, metabolize and excrete certain therapeutic products. Several factors may contribute to these variations, including body composition, hormonal changes, plasma volume, gastric emptying time, plasma protein levels, and cytochrome P450 activity [10–13]. Furthermore, evidence also suggests that the frequency of adverse events (AEs) reported may be higher in women than men, which could be due, at least in part, to potential hormonal effects on physiologic functions [14, 15]. However, by enrolling both male and female subjects in clinical trials, gender-related differences, including drug responses relative to safety and efficacy, can be better identified to more carefully direct clinical decision-making.

**Fig. 1:**
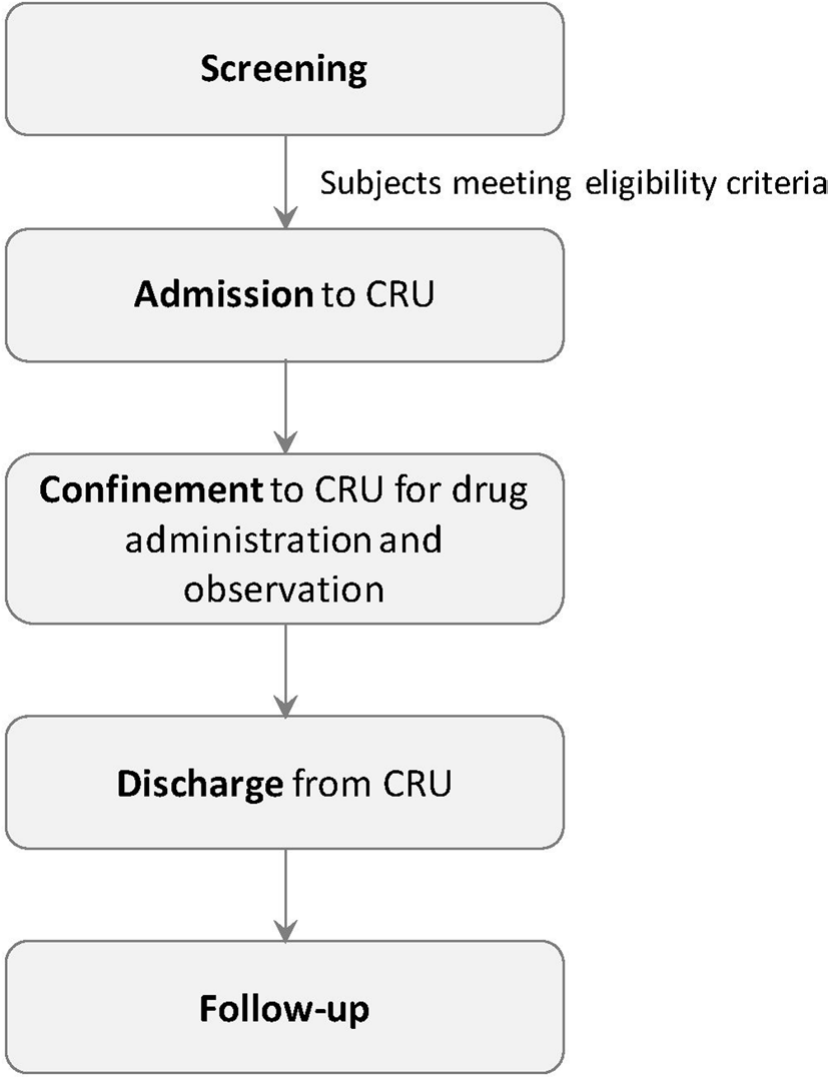
General design of healthy volunteer studies. *CRU* Clinical Research Unit

Phase 1 studies in HVs generally comprise screening of subjects followed by admission of eligible subjects to a clinical research unit, confinement to the clinical research unit until discharge, and follow-up (Fig. 1). These studies have distinct advantages but also raise a variety of ethical questions because HVs are exposed to risks without any expectation or potential of a health benefit. Until recently, however, the true nature of that risk has not been clearly defined. These concerns have prompted re-examination of the underlying rationale for HV studies, the risks involved, and the regulations that govern them.

HVs are recruited by offering financial incentives (i.e. remuneration for their time and trouble). If, as some have suggested, the financial reward is the primary or sole motivation for participation, it raises ethical concerns that study subjects may disregard potential risks or provide false information regarding their health history. However, a systematic review of the reasons that HVs participate in these studies revealed that financial incentives are not the only motivation [16]. Participants cited various other reasons, including a desire to contribute to science or to the health of others, an opportunity to access ancillary healthcare benefits, scientific interest, meeting people, and curiosity. This study further showed that most HVs do carefully consider the risks when making decisions about participation; indeed, risk can be a major deciding factor among HVs [17]. In efforts to investigate potential risks to HVs, a survey of clinicians from the British Pharmacological Society published in 1989 found that < 1% of > 8000 HVs involved in clinical studies over a 12-month period experienced moderately severe AEs, and 0.04% experienced potentially life-threatening AEs [18]. More recently, in a systematic review of 475 HV studies to examine the risk of harm, Johnson and colleagues [19] concluded that Phase 1 HV trials pose a low risk of severe or serious harm to study subjects, reporting that AEs of moderate severity occurred at a rate of 46/1000 participants per monitoring day.

Although the risks to HVs are generally considered to be acceptable, cases that highlight the potential risks have resulted in reforms to the European regulations for HV studies. One case involved an FIH study, conducted in London, United Kingdom in 2006, of an immunomodulatory drug called TGN1412, a novel super agonist anti-CD28 monoclonal antibody that directly stimulates T cells. Six subjects were dosed simultaneously at the no-observed-adverse-effect level (NOAEL), but all 6 rapidly developed severe cytokine release syndrome and acute respiratory distress syndrome requiring intensive supportive care [20]. Although there were no deaths in that case, a similar situation in Rennes, France in 2016 (BIA 10-2474 trial) did result in the death of one HV who received a fatty acid amide hydrolase (FAAH) inhibitor and died 1 week after being hospitalized with neurologic symptoms [21]. As a direct result of the first case, the European Medicines Agency published a guideline in 2007 to emphasize that absolute consideration should be given to characterizing risks and implementing appropriate strategies to mitigate the risks associated with FIH clinical studies [22]. The 2007 guidelines were revised after the case in France [23, 24]. These reforms emphasize that the safety of study subjects (whether patients or HVs) should always be the number one priority. The European Medicines Agency guideline recommends integrated protocols to ensure that relevant animal models are employed, the mechanism(s) of action and PD effects of a drug are well understood, and the starting dose for FIH studies is based on either the minimal anticipated biological effect level or the pharmacologically active dose, which is usually lower than the NOAEL [23].

These important reforms along with a greater understanding and appreciation of the risks to human subjects should ensure that the advantages of HV studies are not overshadowed by unreasonable risks or ethical concerns. In this review, we will focus on the current and future role of HV studies in the development of investigational non-oncology and oncology drugs and examine the design of PK modeling FIH studies in HVs.

## Non-oncology trials

Although the use of HVs in non-oncology Phase 1 clinical trials is relatively safe (defined as a low probability of risk based on preclinical toxicology data and selected starting doses with large safety margins), ethical challenges exist because of the potential risks to which participants are exposed. We present as examples the risks to HVs in studies of 2 classes of non-oncological drugs, namely proprotein convertase subtilisin/kexin type 9 (PCSK9) inhibitors, which lower cholesterol and prevent atherosclerotic cardiovascular disease, and sodium–glucose cotransporter-2 (SGLT-2) inhibitors, which lower serum glucose levels in patients with diabetes and have newly recognized cardiovascular benefits (Table 1).

**Table 1:** Selected trials of non-oncology drugs in healthy volunteers. Source: ClinicalTrials.gov

NCT number	Treatment	Target/MOA	Study design	Outcomes	Enrolled (N)
Completed clinical trials					
NCT01396161	PF-05175157	Acetyl-CoA carboxylase inhibitor	Randomized, double-blind, placebo-controlled	PK, safety	64
NCT00741026	Olanzapine	Muscarinic (M3) receptor antagonist	Randomized, double-blind, placebo-controlled	PK, safety	15
NCT00894322	Exenatide	GLP-1 receptor agonist	Randomized, single-blind	PK, safety	65
NCT01380730	Evolocumab	PCSK9 monoclonal antibody inhibition	Randomized, quadruple-blind, placebo-controlled	PK, safety	629
NCT00924053	Bexagliflozin	SGLT-2 inhibitor	Randomized, quadruple-blind, placebo-controlled	PK, safety	24

GLP-1, glucagon-like peptide 1; MOA, mechanism of action; PCSK9, proprotein convertase subtilisin/kexin type 9; PK, pharmacokinetics; SGLT-2, sodium–glucose cotransporter-2

Inhibitors of PCSK9, a protease that leads to the destruction of low-density lipoprotein cholesterol (LDL-C) receptors, have been developed as adjuncts to diet and maximally tolerated statin therapy for adults with heterozygous familial hypercholesterolemia or clinical atherosclerotic cardiovascular disease requiring additional lowering of LDL-C [25]. Two agents that target and inactivate PCSK9, evolocumab and alirocumab, have been approved by the United States Food and Drug Administration (FDA). Both prevent the destruction of LDL-C receptors, thereby lowering LDL-C levels by 50% to 60%. Regarding inhibitors of PCSK9, monoclonal antibodies have proven to be the most effective [25]. In 3 Phase 1 trials (2 single dose and 1 multiple dose) of alirocumab (REGN727) in 133 HVs, 2 subjects in the single-dose studies had serious adverse events (SAEs); no SAEs were reported in the multiple-dose study [26]. The SAEs were abdominal pain and rectal bleeding in a subject who received placebo, and small bowel obstruction in a subject with an appendectomy history who received alirocumab. Evolocumab (AMG 145) was evaluated in 2 Phase 1, blinded, placebo-controlled, randomized (by dose) trials in HVs, and no SAEs were reported [27].

SGLT-2 inhibitors lower glucose levels by blocking its reabsorption in renal tubules, thereby enhancing excretion of excess glucose [28]. Currently, the FDA has approved 4 SGLT-2 inhibitors: canagliflozin, empagliflozin, ertugliflozin, and dapagliflozin. Canagliflozin and empagliflozin have been evaluated in completed large cardiovascular outcomes trials [29, 30]. These drugs all have similar overall benefit-risk profiles, and they work to reduce HbA1c and fasting glucose levels while occasionally increasing the risk for certain infections. The frequency of AEs associated with SGLT-2 inhibitors has been comparable across the drug class, and risks to Phase 1 participants have remained extremely low at all doses [28]. Ertugliflozin has been tested as part of the VERTIS clinical development program. In a Phase 1 controlled study, the effect of ertugliflozin on cardiac repolarization was examined in 42 HVs [31]. The HVs experienced no clinically significant changes in their electrocardiogram parameters at a supratherapeutic dose (100 mg) of ertugliflozin, and most AEs were of mild severity. Later trials in patients with type 2 diabetes mellitus or stage 3A chronic kidney disease showed that ertugliflozin (5 and 15 mg) could reduce HbA1c levels in both type 2 diabetes mellitus and stage 3A chronic kidney disease cohorts [32].

Similar to that shown in these FIH studies of non-oncology drugs, a meta-analysis of Phase 1 trials conducted at Pfizer dedicated Phase 1 testing sites between 2004 and 2011 showed that in 11,028 HVs who received study drug, most AEs (85%) were mild and only 34 SAEs (0.31%) occurred, with none resulting in life-threatening complications or deaths. Approximately half of all AEs were related to the study drug or to study-related procedures [33]. In another large analysis of Phase 1 trials published between 2008 and 2012, there was a median of zero SAEs and zero severe AEs [19]. The authors concluded that, although recent non-oncological agents in Phase 1 trials may pose mild to moderate risks to HVs, there is a low likelihood of severe harm. In a systematic review of 355 HVs in the Bristol-Meyers Squibb database, which excluded oncology studies, there were no safety concerns regarding SAEs or deaths [34].

These examples highlight the overall favorable safety profile observed in HV studies of investigational non-oncology drugs. Serious or severe AEs are rarely reported. However, as the case of BIA 10-2474 illustrates, there is the potential for non-oncology drugs to cause significant harm, particularly those with neurologic or cardiac effects. In this case, BIA 10-2474 was tested in 2 single-dose, dose-escalation studies at doses up to 100 mg with no safety concerns; however, in a subsequent Phase 1 study, 6 HVs who received multiple daily doses of 50 mg/day over 5 days developed severe neurologic side effects, and one subject went into a coma and died [35]. This study revealed a possible threshold effect of BIA 10-2474 that was not anticipated based on the PK and safety data available at the time. BIA 10-2474 is an FAAH inhibitor that reduces catabolism of endocannabinoids, thereby increasing their concentration in the central nervous system. The endocannabinoids have been implicated in a variety of neurologic conditions such as chronic pain, depression, and anxiety disorders, and a variety of exogenous cannabinoids are approved for use but have some neurologic side effects, such as impaired cognition and motor functions. Administration of an FAAH inhibitor was thought to reduce the risk of those side effects, and several other members of this drug class have been tested clinically and are well tolerated. After intense investigation, it is believed that BIA 10-2474 has off-target effects on several lipases in the brain, which may affect how neurons metabolize lipids. This may have been the cause of the observed toxicity, but a definitive cause has yet to be determined [36]. Nevertheless, this case highlights the potential risk any time a novel agent is tested in humans and emphasizes the importance of rigorous preclinical testing to fully characterize its activity. Furthermore, precise and appropriate safety parameters are necessary to properly screen HVs, to determine clinical trial eligibility criteria for a given therapeutic area, and to carefully monitor HVs during Phase 1 studies to inform dosing decisions.

## Oncology trials

In oncology drug development, early clinical trials have typically not been done in HVs as the investigation of cytotoxic chemotherapy was traditionally only considered ethical in cancer patients [37]. The use of HVs in studies of agents intended for cancer patients may seem paradoxical, yet the principles underlying HV oncology studies are the same as for all HV studies. The perception that it is inappropriate to expose HVs to molecules intended for use in cancer patients stems from the historical use of cytotoxic chemotherapy, with their attendant narrow therapeutic index and potential for lasting DNA damage. However, the emergence of molecularly targeted agents as effective cancer therapies has resulted in opportunities to characterize these molecules in HVs, providing a path forward for increased information gathering without the need for large numbers of cancer patients. Importantly, the use of HVs also allows the circumvention of the traditional ethical dilemma of treating advanced cancer patients with subtherapeutic doses of an investigational drug in order to obtain preliminary safety data. The reduced treatment-related toxicities shown with targeted therapies have led to the reassessment of the potential risks and benefits of HV studies [38]. Following an analysis of Phase 1 clinical trials conducted from 1991 to 2002, which demonstrated a marked reduction in toxic deaths over the time period [39], the FDA issued a statement in favor of HV studies for non-cytotoxic anticancer drugs. Alongside preclinical (e.g. genotoxicity assessments) and clinical considerations (e.g. 1 to 2 doses at most), the FDA cited several reasons to conduct HV clinical trials, including “exploration of bioavailability, reduction of patient exposure to relatively low/ineffective drug doses, and relatively rapid study accrual” [40]. An increase in the number of anticancer Phase 1 clinical trials enrolling HVs has since been observed in recent years [41]. Select clinical trials of oncology drugs in HVs are shown in Table 2.

**Table 2:** Selected trials of oncology drugs in healthy volunteers. Source: ClinicalTrials.gov

NCT number	Treatment	Target/MOA	Study design	Outcomes	Study population	Enrolled (N)
Completed clinical trials						
NCT00658554	ARQ 197	c-MET inhibitor	Randomized, open-label, crossover	PK, safety	Healthy volunteers	24
NCT02474537	INC280 (capmatinib)	c-MET inhibitor	Open-label, parallel-group, two-staged, single-dose	PK, safety	Subjects with impaired hepatic function; healthy subjects with normal hepatic function	31
NCT03154086	GSK3352589	RET growth factor receptor TKI	Randomized, double-blind, crossover, placebo-controlled, single- and repeat-dose escalation	Safety, PK	Healthy volunteers	59
NCT03192111	Entinostat	HDAC inhibitor	Open-label, parallel-cohort, single-dose	PK, safety, tolerability	Adults subjects with mild, moderate, severe renal impairment; healthy volunteers	40
NCT00418626	Nilotinib (AMN107)	TKI	Open-label, single-dose,	PK, impact on hepatic function	Subjects with impaired hepatic function; healthy subjects with normal hepatic function	27
NCT02388620	LEE011 (ribociclib)	CDK4/6 inhibitor	Open-label, parallel-cohort, single-dose	PK, safety	Subjects with impaired hepatic function; healthy subjects with normal hepatic function	30
NCT01764776	LDE225 (sonidegib)	Smo antagonist	Open-label, single-dose	PK, safety	Subjects with impaired hepatic function; healthy subjects with normal hepatic function	33
NCT02050815	MEK162 (binimetinib)	MEK inhibitor	Open-label, single-dose	PK, safety	Subjects with impaired hepatic function; healthy subjects with normal hepatic function	27
NCT02621047	Alectinib	ALK inhibitor	Open-label, single-dose, parallel assignment	Effect of hepatic impairment on PK	Subjects with hepatic impairment; healthy subjects with normal hepatic function	28
NCT01901133	MDV3100 (enzalutamide)	AR antagonist	Open-label, single-dose, parallel assignment	PK, safety	Male subjects with mild or moderate hepatic impairment; male subjects with normal hepatic function	33
Ongoing clinical trials						
NCT02922946 (not yet recruiting)	Entinostat	HDAC inhibitor	Open-label, randomized, single-dose, crossover	Effect of food on PK	Healthy subjects	48
NCT02431481 (ongoing)	LEE011 (ribociclib)	CDK4/6 inhibitor	Open-label, parallel-group, single-dose	PK, safety	Subjects with varying degrees of impaired renal function; healthy volunteers with normal renal function	64
NCT02852239 (ongoing)	Dabrafenib	BRAF inhibitor	Open-label, single-dose	PK, safety	Subjects with impaired renal function; healthy subjects with normal renal function	~ 32
NCT02852239 (ongoing)	Dabrafenib	BRAF inhibitor	Open-label, single-dose	PK, safety	Subjects with impaired hepatic function; healthy subjects with normal hepatic function	~ 32

ALK, anaplastic lymphoma kinase; AR, androgen receptor; BRAF, v-raf murine sarcoma viral oncogene homolog B1; CDK4/6, cyclin-dependent kinases 4 and 6; HDAC, histone deacetylase; MOA, mechanism of action; PK, pharmacokinetics; TKI, tyrosine kinase inhibitor

**Fig. 2:**
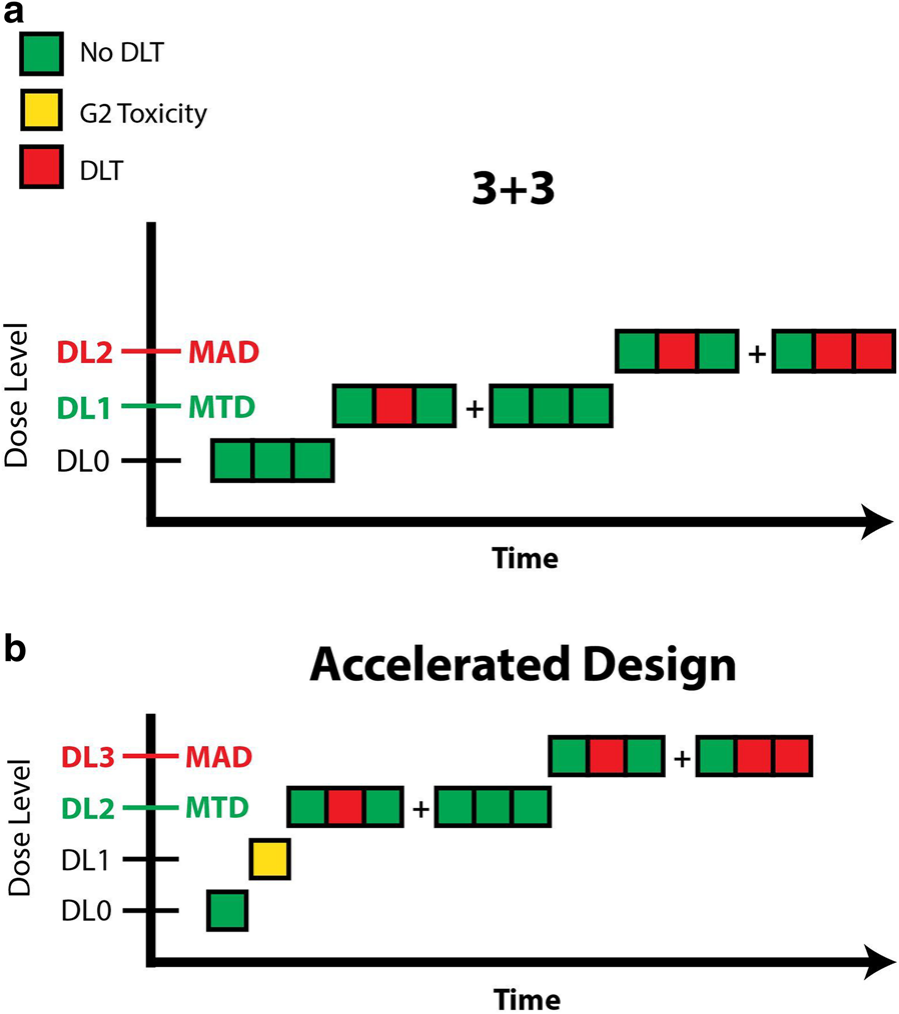
Traditional (**a**) and modified (**b**) first-in-human study designs. *DL* dose level, *DLT* dose-limiting toxicity, *G2* grade 2, *MAD* maximum administered dose, *MTD* maximum tolerated dose

Traditional oncology FIH trials use a modified version of the up-and-down method created in 1948 by Dixon and Mood [42]. In the traditional 3 + 3 Phase 1 design, a minimum of 3 participants is studied at each dose level (Fig. 2a). If none of the 3 participants experiences a dose-limiting toxicity (DLT), the next group of 3 participants is enrolled into the subsequent highest dose level. If one of the 3 participants experiences a DLT, up to 3 additional participants are enrolled for a total of 6 participants. When DLTs are observed in at least 2 participants out of either 3 or 6 participants, the maximum administered dose is reached and additional participants are enrolled in the next lower dose level (the maximum tolerated dose). The maximum tolerated dose is defined as the dose level at which none or 1 of 6 participants (0% to 17%) experiences a DLT. In the 3 + 3 design, accrual is suspended after enrollment of each cohort of 3 participants and resumed when all 3 participants have cleared the DLT period. The dose is increased in each subsequent cohort using a modified Fibonacci sequence in whichever higher escalation steps have ever decreasing relative increments (e.g. dose increases of 100%, 65%, 50%, 40%, and 30% thereafter).

The traditional 3 + 3 design has many limitations, including long delays in accrual, replacement of nonevaluable patients, and limited characterization of PK given the small sample sizes. In addition, it can be difficult to determine whether an AE is related to the investigational drug or to a symptom of the underlying metastatic cancer. Moreover, due to the conservative nature of the dose-escalation scheme, many patients are exposed to subtherapeutic doses of the study drug, thus raising the ethical question of whether it is appropriate to knowingly expose patients with advanced cancer to ineffective doses of experimental therapies. Some investigators have thus modified the traditional design to enroll only 1 patient per cohort and conduct sequential 100% dose escalations until a drug-related grade 2 toxicity is observed, at which point the traditional 3 + 3 design and modified Fibonacci dose escalations commence. This has been termed the “accelerated titration design” (Fig. 2b). Another modification, the “rolling six,” has been proposed as a means of accelerating FIH cancer trials, albeit with a slight increase in the number of patients required [43].

Conducting FIH trials of oncology drugs in HVs can address many of the issues raised with the 3 + 3 design or its variants. Accrual is generally very rapid with all subjects being enrolled on the same day, nonevaluable subjects are rare, and sample size can be increased with no increase in time to conduct the trial. Also, the relationship of AEs to study drug can generally be clearly ascertained due to the otherwise healthy nature of the study subjects. Perhaps most importantly, patients with advanced cancer are not exposed to subtherapeutic doses of experimental therapies. The main disadvantage of conducting FIH trials in HVs is an inability to assess the PD effects of the drug if the molecular target is unique to the cancer cell (e.g. a tumor-specific mutated protein). Furthermore, it is obviously not possible to examine antitumor activity in HVs.

Two issues typically are considered when deciding whether it is appropriate to conduct an FIH study of an oncology drug in HVs: potential for genotoxicity and predicted starting dose. Genotoxicity is defined as the property of a chemical agent to damage DNA, potentially leading to carcinogenesis. HV studies require completion of in vitro and in vivo genotoxicity studies, whereas genotoxicity study requirements are generally waived for cytotoxic drugs, which are already known to be genotoxic. Thus, when considering whether to conduct studies in HVs, investigators will need to consider the time and cost to conduct genotoxicity studies and consider whether the specific mechanism of the investigational drug is likely to be genotoxic. The predicted starting dose also plays a crucial role in determining feasibility of an FIH HV study. As previously noted, the starting dose for an FIH HV study is generally 1/10 of the rodent NOAEL. In contrast, the starting dose for cytotoxic drugs is generally 1/10 of the rodent severely toxic dose. If the preclinical efficacious dose is equal to or less than the NOAEL and the mechanism of the drug is predicted to be non-genotoxic, a strong case can be made to conduct the FIH trial in HVs. If the preclinical efficacious dose far exceeds the NOAEL or approaches 1/10 of the severely toxic dose, it is still possible to conduct the FIH trial in HVs, albeit simply to characterize the PK of the molecule. It is important to note that the above considerations apply equally to small molecules, monoclonal antibodies, cell therapies, and other emerging modalities such as RNA therapeutics. Indeed, HV studies have been conducted, for example, with anti-colony-stimulating factor 1 (CSF1) and anti-CSF1R antibodies [44].

## Characterizing the clinical pharmacology of anticancer drugs in healthy volunteers

Although most studies in an oncology drug development program are designed to characterize the safety and efficacy of the molecule, an even greater number of studies are conducted to characterize the clinical pharmacology of the molecule. Clinical pharmacology studies for anticancer drugs have multiple objectives and designs that lend themselves to being conducted in HVs (Table 3), and they differ greatly from typical FIH studies conducted in cancer patients who have exhausted all lines of therapy in the era of cytotoxic chemotherapy [39]. In fact, HVs may be a better population for assessing the PK of a novel drug candidate because performance status can contribute to differences in PK parameters. Although representative patient populations are essential for appropriate dose selection, much can be learned from clinical pharmacology studies conducted in HVs. Such studies typically utilize small cohorts, with appropriate controls, to provide specific information about PK, drug metabolism, food effects, potential drug–drug interactions, effects of hepatic and renal impairment, and other pharmacologic parameters vital for clinical decision making. Studies in HVs are also being used to demonstrate the similarity of biosimilars to established anticancer biologic agents.

**Table 3:** Selected published trials of oncology drugs in healthy volunteers sorted by study objective

NCT number, PMID number	Treatment	Target/MOA	Study population/study design	Study objective/study endpoints	Enrolled (N)
Mass balance					
NCT01674322, 25488930 [47]	[^14^C]-Ibrutinib	Bruton tyrosine kinase	Single center, open-label, single-dose study in healthy males	PK of total radioactivity, parent drug and metabolites (plasma, urine, feces) based on CYP2D6 genotype	6 (2 CYP2D6 poor metabolizer subjects)
NCT01711762, 26451002 [46]	[^14^C]-Cobimetinib	MEK inhibitor	Single center, open-label, nonrandomized study in healthy males	PK of total radioactivity, parent drug and metabolites (plasma, urine, feces)	6
Bioequivalence					
N/A, 26272586 [52]	Ceritinib	ALK inhibitor	Two randomized, open-label, 2-period, crossover studies in healthy subjects	Changes in bioavailability with low- or high-fat meal, or light snack compared to fasting conditions	40 (28, 12)
N/A, 25643050 [53]	Erlotinib	EGFR inhibitor	Two single center, open-label, crossover studies (two and three sequence) in healthy subjects	Changes in bioavailability when coadministered with ranitidine or omeprazole	48 (24 each)
N/A, 28776077 [57]	Abiraterone Acetate	CYP17 inhibitor	Single center, open-label, fixed treatment sequence, 3-period, single-dose, crossover study in healthy males	Changes in bioavailability with novel formulation under both fasted and fed conditions compared with historical controls	11
Biosimilarity					
NCT01603264, 25041377 [80]	Trastuzumab (PF-05280014, EU-, or US-approved trastuzumab)	HER2 inhibitor	Single center, double-blind, parallel group, single-dose, 3-arm, active comparator trial in healthy subjects	Establish 3-way clinical PK similarity of PF-05280014 to both EU- and US-trastuzumab formulations, and of EU- to US-trastuzumab	101
NCT01608087, 28651442 [59]	Bevacizumab, (BI 695502, US-, or EU-approved bevacizumab)	VEGF inhibitor	Two center, randomized, single-blind, single-dose, two-stage, 3 parallel-arm, active-comparator trial in healthy subjects	Establish 3-way clinical PK similarity to enable use of just one reference product for a Phase III biosimilarity trial	91
Drug–drug interaction					
NCT01147055, NCT01149785, 26381275 [81]	Crizotinib	ALK inhibitor	Two single center, open-label, 2-period, 2-treatment, 1-sequence, single-dose, crossover studies in healthy subjects	Estimate the effects of multiple doses of ketoconazole and rifampin on single-dose PK	30 (15 each)
N/A, 20702754 [66]	Nilotinib	BCR/ABL inhibitor	Two single center, open-label, 2-period, 1 sequence, single-dose crossover studies in healthy subjects	Estimate the effects of multiple doses of ketoconazole and rifampin on single-dose PK	15 (rifampin) 26 (ketoconazole)
Organ impairment					
NCT01298063, 24906422 [68]	Afatinib	EGFR inhibitor	Single center, open-label, single-dose study comparing mild (n = 8) and moderate hepatic impairment (n = 8) to matched healthy controls (n = 16)	Compare relative drug exposure between subjects with hepatic impairment and matched healthy controls, as measured by AUC_0−∞_ and C_max_	32
NCT02096718, 27436099 [69]			Single center, open-label, single-dose study comparing moderate (n = 8) and severe renal impairment (n = 8) to matched healthy controls (n = 14)	Compare relative drug exposure between subjects with renal impairment and matched healthy controls, as measured by AUC_last_ and C_max_	30
Pharmacokinetic/pharmacodynamic relationships					
N/A, 28660498 [70]	Degarelix	GnRH antagonist	Single center, double-blind (open-label for moxifloxacin) randomized, crossover study in healthy males	Compare the effect of single-dose degarelix, placebo, and moxifloxacin on the QT interval	80
NCT00406406, 22884766 [64]	Bosutinib	Src/Abl inhibitor	Single-center, randomized, double-blind, placebo-controlled, single-ascending dose, sequential-group study in healthy subjects	Assess the safety, tolerability, and PK with ascending doses to support further clinical pharmacology assessment	48
N/A, 20376000 [73]	Sunitinib	PDGFR/VEGFR/KIT/FLT3/CSF1R/RET inhibitor	Single center, open-label, multiple dose study in healthy subjects	To develop a PD/PK model to investigate the concentration-effect relationship between drug and biomarkers (blood pressure, VEGF-A, VEGF-C and sVEGFR-2)	12

ALK, anaplastic lymphoma kinase; AUC, area under the curve; Cmax, maximum concentration; EGFR, endothelial growth factor receptor; GnRH, gonadotropin-releasing hormone; MOA, mechanisms of action; N/A, not available; PD, pharmacodynamics; PDGFR, platelet-derived growth factor receptor; PK, pharmacokinetics; VEGF, vascular endothelial growth factor; VEGFR, VEGF receptor

Clinical pharmacology studies employ a wide range of designs. For example, the crossover study design is widely used to compare different formulations of a drug. A reference formulation is initially given to the subject, followed by a washout period and the administration of an investigational formulation. Crossover studies reduce the required number of subjects and limit potential sources of variation or confounding [45]. Deviations from crossover studies include matched control studies and randomized controlled studies, and these are justified on the basis of a particular study objective and/or the level of evidence required. Complete characterization of absorption, distribution, metabolism, and elimination can also be accomplished by administering a radiolabeled drug to HVs (n < 10) in a biospecimen collection-focused mass balance study design [23, 46–48].

Determination of bioequivalence is an industry standard approach to quantify whether the maximum concentration, time to maximum concentration, and area under the concentration–time curve (AUC_t_ and AUC_0−∞_) of a new drug formulation is within the 80% to 125% range of the 90% confidence interval of a reference formulation (historical data or data collected on study), which is required to demonstrate that the formulations are bioequivalent [49]. Bioequivalence studies are especially important for understanding oral formulations, which have become a standard drug delivery method in oncology during the era of targeted therapies (e.g. tyrosine kinase inhibitors) [50]. Such studies can optimize the drug delivery by investigating several experimental formulations and determining the extent of absorption with and without food (i.e. food effect studies) [51, 52] or with gastric pH-lowering agents (e.g. H_2_-receptor antagonists, proton-pump inhibitors) [53, 54]. Bioequivalence studies typically use randomized, open-label, single-dose, Phase 1 designs that can enroll HVs. In recent investigations of abiraterone acetate, a CYP17 inhibitor approved for metastatic prostate cancer, HV studies have been used to investigate bioequivalence to a reference formulation with smaller doses via exploitation of food effect or optimized formulations [55–57]. Other recent HV studies, specifically studies investigating bevacizumab formulations [58–61], have incorporated blinded, randomized, single-dose, parallel group designs with at least 30 subjects per group.

HVs have recently been enrolled in studies evaluating biosimilars to originator biologics such as trastuzumab [62]. Studies in HVs can be used to assess PK bioequivalence and to compare the immunogenicity of a biosimilar with that of the originator biologic [63]. In this setting, HVs are ideal study subjects because, unlike cancer patients, they have a fully intact immune system.

HV studies are also frequently used to measure metabolism and elimination and assess factors that can affect metabolism and elimination. For example, drug–drug interaction studies determine whether specific agents co-administered with the drug in question can affect its metabolism. Drugs metabolized by the same cytochrome P450 (CYP) enzymes in the liver often exhibit drug–drug interactions. HV drug–drug interaction studies typically utilize a crossover design, quantifying the PK parameters of the study drug with and without a known enzyme inhibitor (e.g. ketoconazole and CYP3A4) or inducer (e.g. rifampin and CYP3A4) [64–66]. The effects of hepatic and renal impairment on clearance and AUC have also been assessed in studies of HVs. However, because a crossover design is not feasible for these studies, subjects with the specified organ impairment are usually matched with healthy controls to generate the appropriate comparisons [67–69].

PD endpoints have also been incorporated into HV studies of anticancer agents, specifically in those focusing on dose-dependent toxicity or PD effect. A common example is studies investigating drug-induced QT prolongation, which utilize randomized crossover study designs with the added inclusion of a positive control (e.g. moxifloxacin) [70, 71]. Dose-escalation studies with short-term dosing schemes have also been used to evaluate maximum tolerated doses in HVs [72] and dose-dependent changes in PD biomarkers, which serve as a surrogate for PD effects and offer insight into the drug’s mechanism of action [73]. However, HV studies investigating dose-dependent toxicity and biomarker-driven PD effects usually do not provide sufficient evidence of safety or efficacy, and additional studies in cancer patients are often needed to fully characterize the profile of the drug.

Data from HV studies are also currently being incorporated into population PK models to analyze the effects of patient-specific characteristics (e.g. weight, age, genotype) on PK parameters (e.g. volume of distribution or clearance). Some published models have incorporated only HV study data [74], whereas others have included data from both HVs and cancer patients (Table 4) [75, 76]. Although population PK models can be useful, the intent of such models must be well-defined, especially for prediction of patient-specific doses. Differences in PK parameters between HVs and cancer patients should be assumed and then tested as a covariate in model development unless proven otherwise. For example, a recent population PK analysis of cabozantinib demonstrated that patients with medullary thyroid carcinoma had an approximate 93% increase in clearance relative to HVs, leading to 40% to 50% lower predicted steady-state plasma concentrations [77]. As a result, the FDA-approved dose for medullary thyroid carcinoma is 140 mg compared with only 60 mg for renal cell carcinoma [77]. This example highlights the potential limitations of population PK data based solely on HVs.

**Table 4:** Selected population pharmacokinetic analyses of anticancer drugs with the inclusion of healthy volunteers

PMID number	Treatment (MOA)	Total studies included	Purpose of population pharmacokinetic model	Key findings	Number of subjects
23834452 [74]	Axitinib (VEGFR1, 2, and 3 inhibitor)	10 (all Phase 1)	Estimate population parameters, evaluate effect of food, formulation, demographics, organ function, metabolic genotype	Volume of distribution increased with body weight, but no impact on drug exposure warranting dose modification	337 (all healthy)
28833380 [82]	Selumetinib (MEK inhibitor)	10 (all Phase 1)	To determine whether the same model is suitable for all formulations and fed conditions and to understand differences in absorption and relative bioavailability with each study	Confirmed previous findings on bioavailability; additional covariates discovered, none requiring dose modification	346 (all healthy, includes subjects with organ impairment)
26879594 [75]	Lenvatinib (VEGFR1, 2, and 3, PDGFRβ, RET inhibitor)	15 (12-Phase 1, 2-Phase 2, 1-Phase 3)	To characterize the PK profile, and identify factors to explain interindividual PK variability in healthy subjects and patients with cancer	No clinically relevant covariates requiring dose adjustment (including demographics, biomarkers, co-administered agents, organ function)	779 (196 healthy, 583 patients with cancer)
26898300 [76]	Sonidegib (Smo antagonist)	5 (4-Phase 1, 1-Phase 2)	To develop a structural PK model in healthy subjects and in patients with cancer, characterize covariate effects, and determine sources of variability	High-fat meals increased bioavailability fivefold, healthy volunteers had threefold higher clearance, no dose adjustments needed based on patient characteristics (patients with cancer)	436 (85 healthy, 351 patients with cancer)
29687244 [77]	Cabozantinib (VEGFR2, RET, c-MET inhibitor)	9 (3-Phase 1, 2-Phase 2, 4-Phase 3)	To analyze the combined PK data from different patient populations and HVs to assess the effect of cancer type, formulation and dose	Small to moderate effect of demographic covariates on apparent clearance (CL/F); Patients with MTC had higher CL/F compared with HVs and other cancer types	1534 (140 healthy, 1394 patients with cancer)

HV, healthy volunteers; MOA, mechanisms of action; MTC, medullary thyroid cancer; PDGFR, platelet-derived growth factor receptor; PK, pharmacokinetics; VEGFR, vascular endothelial growth factor receptor

## Conclusion and future directions

When tailored to specific study objectives, HV studies are beneficial to both manufacturers and patients alike and can be applied to both non-oncology and oncology drug development. Enrollment of HVs not only increases study accrual rates for single- and multiple-dose PK endpoint-driven studies but also alleviates the ethical concern of enrolling patients with advanced disease in a short-term study at subtherapeutic doses when other studies (e.g. Phase 2 or Phase 3 studies) may be more appropriate for the patient. The use of HVs for FIH studies of non-oncology drugs is generally safe, and SAEs are rare, although examples of severe toxicity have been reported. Although the use of HVs in the clinical development of oncology drugs is more limited, it is nonetheless useful for evaluating clinical pharmacology and establishing an appropriate starting dose for studies in cancer patients. A thorough assessment of toxicity and pharmacology throughout the drug development process is critical to ensure the safety of HVs. With the appropriate safeguards, HVs will continue to play an important role in future drug development.

Over the past several years, a fundamental shift has occurred in the clinical research community to engage study participants as partners in the design and conduct of clinical research as opposed to engaging them purely as subjects from whom data are collected and outcomes measured. Embracing participants as collaborators has been driven by many factors, including poor patient trust of clinical research and the onerous nature of many clinical trial protocol procedures and follow-up [78]. Groups such as the Patient-Centered Outcomes Research Institute, which recently launched the National Patient-Centered Clinical Research Network (PCORnet), have systematically brought together patients, clinicians, researchers and healthcare system leaders to create policy, infrastructure and acceptance for evidence generation through large simple pragmatic trials that benefit from participants as collaborators.

In 2018, the Clinical Trials Transformation Initiative published information about the potential advantages of patient engagement. Patient engagement efforts can result in enhanced clinically relevant hypotheses, assist in identifying relevant measurements for patient outcomes, limit time and emotional burden for research participation, and lead to improvements in recruitment and perhaps more importantly retention in clinical studies [79]. Although many aspects of patient engagement with researchers are motivated by specific interests in their own disease or that of a family member, many of the learnings from recent patient engagement efforts are directly applicable to HV studies. For example, engaging patient groups early in the clinical trial process and investing in their education, not only in the science of their disease but also in clinical trial design, can contribute to identification of the optimal study population, ultimately resulting in more efficient accrual and shortened timelines.

## Abbreviations

AE: adverse event
AUC: area under the curve
CSF: colony-stimulating factor
DLT: dose-limiting toxicity
FAAH: fatty acid amide hydrolase
FDA: US Food and Drug Administration
FIH: first-in-human
HV: healthy volunteer
LDL-C: low-density lipoprotein cholesterol
NOAEL: no-observed-adverse-effect level
PD: pharmacodynamics
PK: pharmacokinetics
PCSK9: proprotein convertase subtilisin/kexin type 9
SAE: serious adverse event
SGLT-2: sodium–glucose cotransporter-2
